# Role of immunostimulatory deoxycytidylate-phosphate-deoxyguanylate (CpG) motifs in oral bacteria associated with oral diseases

**DOI:** 10.1080/20002297.2025.2486639

**Published:** 2025-04-07

**Authors:** Pisit Charoenwongwatthana, Oslovenya S. Caroline, Halah Ahmed, Jamie Coulter, Chien-Yi Chang

**Affiliations:** a School of Dental Sciences, Faculty of Medical Sciences, Newcastle University, Newcastle upon Tyne, UK; b Department of Oral Medicine and Periodontology, Faculty of Dentistry, Mahidol University, Bangkok, Thailand; c Faculty of Dentistry, Universitas Indonesia, Jakarta, Indonesia

**Keywords:** CpG, immunostimulatory, oral bacteria, genome, endodontic diseases, periodontal diseases, caries

## Abstract

**Background:**

CpG oligodeoxynucleotide motifs in bacterial DNA with composition variations exhibit potent immunostimulation. The effect of different compositions in oral infections is unclear. This study aims to investigate CpG motifs in bacteria associated with endodontic diseases, periodontal diseases, and dental caries to elucidate their influence on host immune response.

**Methods:**

Fifty oral bacterial genomes were selected for *in silico* analysis to determine GC% content and CpG motif frequency in each genome. The relationships between GC% content, CpG motif frequency, and genome size were assessed using correlation analysis. Normalisation of immunostimulatory sequences was implemented to enable unbiased comparison of frequency counts among bacteria.

**Results:**

Sixty percent of bacteria exhibited medium GC% content (*Mdn* = 44), with no significant difference among bacteria associated with these diseases (*p* = 0.66). A positive correlation between GC% content and CpG motif frequency, as well as genome size and CpG motifs frequency was observed. A higher-than-mean of the human immunostimulatory motif (GTCGTT, 7/14) and the mice/rabbits immunostimulatory motif (GACGTT, 9/14) was observed in core endodontic microbiota.

**Conclusion:**

CpG motifs in oral bacteria might drive disease progression through host immunomodulation. Variation in bacterial CpG motifs suggests targeting these motifs offers a promising therapeutic intervention.

## Introduction

The oral microbiome consists of a diverse community of microorganisms residing within the oral cavity, including bacteria, fungi, viruses, and protozoa, with bacteria comprising the majority [1]. Under healthy physiological conditions, a symbiotic relationship exists between the bacteria and host cells. However, ecological changes can disrupt this balance of microbiota, leading to an overgrowth of pathogenic bacteria. Consequently, this imbalance develops inflammatory diseases such as endodontic and periodontal diseases [2]. Endodontic diseases arise from bacterial infection of the dental pulp space, which may result from dental caries, trauma, iatrogenic injury, or periodontal diseases [3]. Although bacterial infection is a causative factor, only the specific bacteria niches are associated with oral diseases. For instance, *Porphyromonas endodontalis*, *Olsenella uli*, and *Propionibacterium spp*. are the dominant members of endodontic infections [3,4]. *Porphyromonas gingivalis*, *Treponema denticola*, and *Tannerella forsythia*, known as the red complex, are recognised as crucial pathogens contributing to severe periodontal disease [5], while *Streptococcus mutans* and Lactobacilli are key culprits linked to the aetiology of dental caries [6]. The varying locations of these bacteria niches within the oral cavity, along with their specific nutrient and growth requirement, contribute to the development of different diseases. However, it is important to note that some bacteria, such as *P. gingivalis*, *Fusobacterium nucleatum*, and *Prevotella intermedia*, are causative pathogens that contribute to both endodontic diseases and periodontal diseases, highlighting bacterial versatile adaptation to survive in different niches and the complex interplay between host and pathogen in these oral infections [7].

Generally, a key mechanism by which bacteria initiate disease pathogenesis is successful colonisation and proliferation in a susceptible host tissue. Host immune responses can be triggered through the recognition of pathogen-associated molecular patterns (PAMPs) such as lipopolysaccharide (LPS) in Gram-negative bacteria and lipoteichoic acid (LTA) in Gram-positive bacteria, leading to inflammation and tissue damage [4]. These bacterial components activate the Toll-like receptors (TLRs) signalling pathway in host cells, thereby inducing the release of proinflammatory cytokines, e.g. interleukin (IL)-1β and IL-6. This signalling cascade ultimately promotes the recruitment of immune cells, e.g. neutrophils and macrophages, to the site of infection [8,9]. Subsequently, the hostile interactions between host immune cells and bacterial infections result in further tissue disruption, which is necessary to prevent the spreading of pathogens to other sites and create the space for specialised cell infiltration for subsequent healing processes, but is damaging to the local environment. This inflammatory process could manifest as pulp necrosis, periodontal tissue destruction, and bone resorption in endodontic and periodontal diseases [10].

Besides bacteria-associated molecules, bacterial DNA can also serve as a virulent factor, initiating disease pathogenesis. Following bacterial cell death, residual bacterial DNA can remain and activate inflammatory responses [11]. Bacterial DNA contains short sequences of cytosine linked to guanine by phosphodiester bond in the 5’ to 3’ direction, known as deoxycytidylate-phosphate-deoxyguanylate (CpG) motifs, which have been seen as potent immunostimulants. These motifs as hexamers consist of an unmethylated CG dinucleotide flanked by two purine residues on the 5’ end and two pyrimidine residues on the 3’ end [12]. The immunostimulatory activity of CpG motifs is associated with the context of motifs [13]. The specific sequence GTCGTT has been identified as the optimal human immunostimulatory motif (HIM), while GACGTT is optimal mice/rabbits immunostimulatory motif (MRIM) [14]. The immunostimulatory mechanism of CpG motifs is to bind to the Toll-like receptor 9 (TLR-9), activating the nuclear factor-κB (NF-κB) pathway. The activation triggers an inflammatory response by promoting the transcription of pro-inflammatory interleukin genes, including IL-1β, IL-6, IL-12, and tumour necrosis factor (TNF-α) [15].

TLR-9 signalling stimulated by bacterial CpG motifs is involved in the development of endodontic diseases and periodontal diseases [16]. The activation through the TLR-9 signalling pathway has been shown to induce periodontal bone loss in *P. gingivalis*-induced periodontitis in mice by increasing levels of IL-6, TNF-α, and osteoclast activity [17]. Additionally, odanacatib, a Cathepsin K inhibitor, has been demonstrated to disrupt the interaction of TLR-9 and bacterial CpG motifs. This impairment in TLR-9 signalling results in a diminished inflammatory response and reduced bone resorption in endodontic diseases in mice [18]. This highlights a crucial role of bacterial CpG motifs in the interaction of immune response and contributing to oral diseases.

The immunostimulatory capability of CpG motifs through TLR-9 activation has shown a positive correlation with the GC% content in gut bacterial genomes [19]. Moreover, bacterial CpG motifs have been investigated in several bacterial communities involved in inflammatory diseases, such as inflammatory bowel disease and bacterial sexually transmitted disease, and have shown their potential to influence host immunity [19,20]. The GC% content in the bacterial genome varies in species and strains enduring bacterial capacities of adaptation to different surrounding environments to thrive in specific niches [21]. Since endodontic diseases, periodontal diseases, and dental caries have distinct ecosystems and bacterial niches [22–24], we hypothesise that the likelihood of CpG motifs in the oral bacterial genome with different GC% content may correlate to their pathogenicity in different oral diseases through varying levels of immunostimulation. In this study, we aim to identify potent CpG motifs in oral bacterial genomes and explore the immunostimulatory potential of these motifs in relation to endodontic diseases, periodontal diseases, and dental caries.

## Materials and methods

### Identification of oral bacteria associated with oral diseases

Fifty strains from 35 oral bacterial species were selected from the expanded Human Oral Microbiome Database (eHOMD, https://www.homd.org). This selection comprised 14 bacterial species associated with endodontic diseases, 4 with periodontal diseases, 10 with dental caries, and 7 that contribute to more than one of these diseases (referred to as multiple oral diseases in this study). These bacteria were selected as some of the most highly prevalent and pathogenic in relation to oral diseases [7,24–28]. The genome sequences of 50 strains were acquired from the National Center for Biotechnology Information (NCBI, https://www.ncbi.nlm.nih.gov/) database in FASTA format. The list of oral bacteria and their genomic information was compiled in supplementary file 1.

### Bioinformatic analyses of CpG motifs

*In silico* analyses were conducted using SnapGene software (GSL Biotech LLC, Boston, MA, USA) to identify CpG motifs and evaluate GC% content in selected bacterial genomes. The analysis was conducted on hexamers to evaluate all 256 possible CpG motif sequence (NNCGNN) arrangements. Briefly, DNA consists of different combinations of nucleotide bases: adenine and guanine (A and G, purines designated as R), and thymine and cytosine (T and C, pyrimidines designated as Y). Therefore, 16 distinct CpG motif arrangements were identified based on this classification. To further identify experimentally immunostimulatory motifs, the frequency of GTCGTT (optimal sequences for humans) and GACGTT (optimal sequences for mice and rabbits) and GC% of different oral bacteria associated with endodontic diseases, periodontal diseases, dental caries, and multiple oral diseases were evaluated for comparison. CpG frequency was normalised by dividing the value of CpG frequency counts with the value of GC% content and genome size (megabase, Mb) to account for variations due to different genome sizes and base compositions.

### Statistical analyses

Statistical analyses were conducted using GraphPad Prism 10 (GraphPad Software Inc., San Diego, CA, USA). The normality of data distribution was determined using the Shapiro–Wilk test. The Kruskal–Wallis test was employed to compare the GC% content among groups. Spearman’s rank correlation coefficient was used to assess the relationships between CpG frequency with GC% content and CpG frequency with genome size. A p-value below 0.05 was considered statistically significant in this study.

## Results

### GC% content in oral bacterial genomes associated with oral diseases

**Figure 1.**
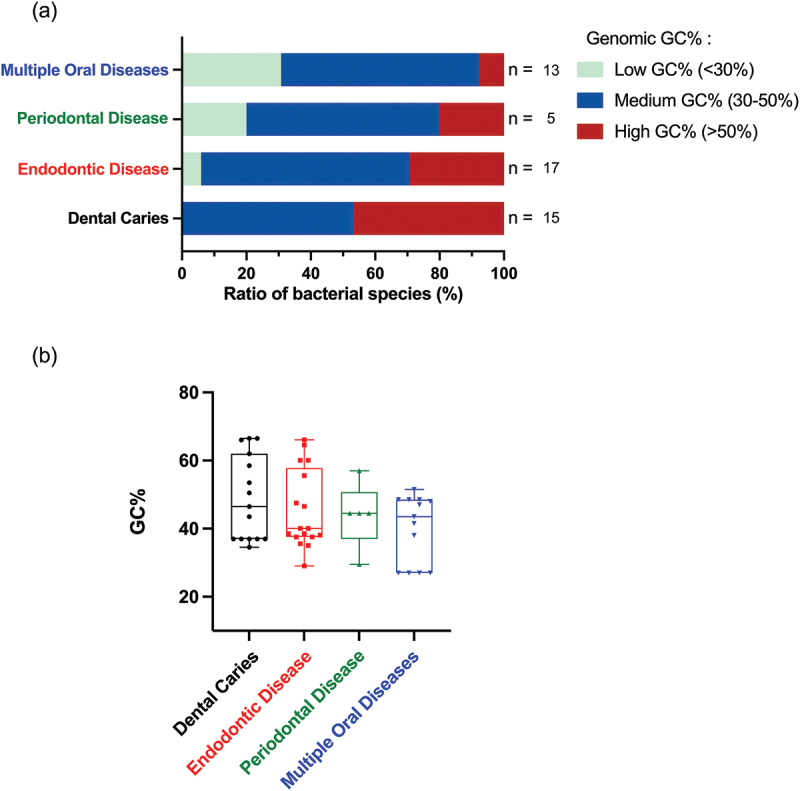
A comparison of GC% content of bacteria across four disease groups: dental caries, endodontic diseases, periodontal diseases, and multiple oral diseases. (a) Most bacteria in our study (60%) exhibit medium GC% content. Bacteria associated with dental caries present medium to high GC% content, whereas bacteria in the remaining groups show low to medium GC% content. (b) The median GC% content of bacteria in each group was 46.5 for dental caries, 40 for endodontic disease, 44.5 for periodontal disease, and 43.5 for multiple oral diseases. No significant difference in GC% content was observed among the groups, *p* = 0.66.

Genome analysis was performed to determine the GC% content and genome size in selected oral bacteria (Supplementary file 1). The GC% content can be categorised into three groups: low (<30%), medium (30–50%), and high (>50%). In our study, 60% of the bacterial strains exhibit medium GC% content (*Mdn* = 44), with a proportion ratio of 64.71% for endodontic diseases, 60% for periodontal diseases, 53.33% for dental caries and 61.54% for multiple oral diseases (Figure 1a). Only the bacteria associated with dental caries presented medium to high GC% content.

*S*. *maltophilia*, associated with dental caries, showed the highest value of GC% (66.5%), whereas *F. nucleatum*, the bacterium associated with multiple oral diseases, displayed the lowest value (27%). The GC% content in bacteria for each group was further analysed to compare group differences. No significant difference was found in GC% content among dental caries (*Mdn* = 46.5), endodontic disease (*Mdn* = 40), periodontal disease (*Mdn* = 44.5), and multiple oral diseases (*Mdn* = 43.5), *H*(3) = 1.61, *p* = 0.66 (Figure 1b). Our analysis revealed variations in GC% content among bacteria associated with different oral diseases.

### Correlation between frequency of CpG motifs with GC% content and genome size

**Figure 2.**
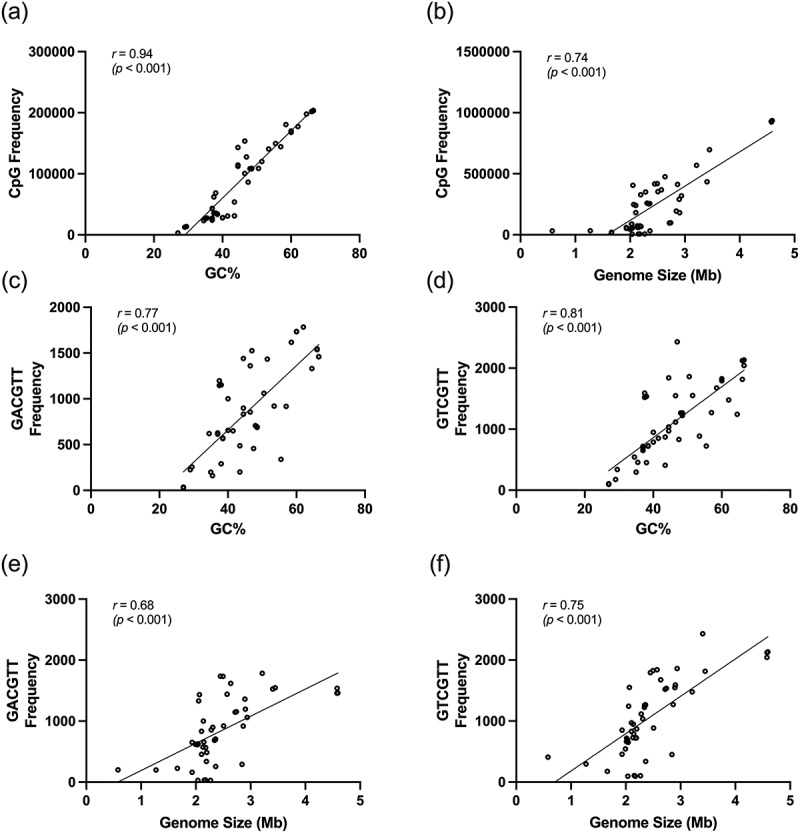
The correlation between CpG frequency with GC% content and genome size are shown. (a) The frequency of CpG motifs increased with rising GC% content (*r* = 0.94). (b) CpG motifs frequency also positively correlated with the genome size (*r*  = 0.74). (c) The frequency of MRIM (GACGTT) increased with rising GC% content (*r* = 0.77). (d) A positive correlation was also observed with MRIM (GACGTT) frequency and genome size (*r* = 0.81). (e) The frequency of HIM (GTCGTT) increased with rising GC% content. (f) The frequency of HIM (GTCGTT) also positively correlated with genome size (*r* = 0.75).

Analysis of CpG motifs revealed a positive correlation between the frequency of the CpG hexamer motif and genomic GC% content, *r(*48) = 0.94, *p* < 0.001 (Figure 2a). A positive correlation was also observed between the frequency of the CpG motifs arrangements and genome size, *r(*48) = 0.74, *p* < 0.001 (Figure 2b). Furthermore, frequencies of both MRIM (GACGTT) and HIM (GTCGTT) increased with the risen genomic GC% content, *r(*48) = 0.77, *p* < 0.001 and *r(*48) = 0.81, *p* < 0.001, respectively (Figure 2c,d). These frequencies also increased with genome size, *r(*48) = 0.68, *p* < 0.001 and *r(*48) = 0.75, *p* < 0.001, respectively (Figure 2e,f). Therefore, positive correlations were anticipated between CpG frequency, increased GC% content, and genome size.

### Normalised CpG frequency

**Figure 3.**
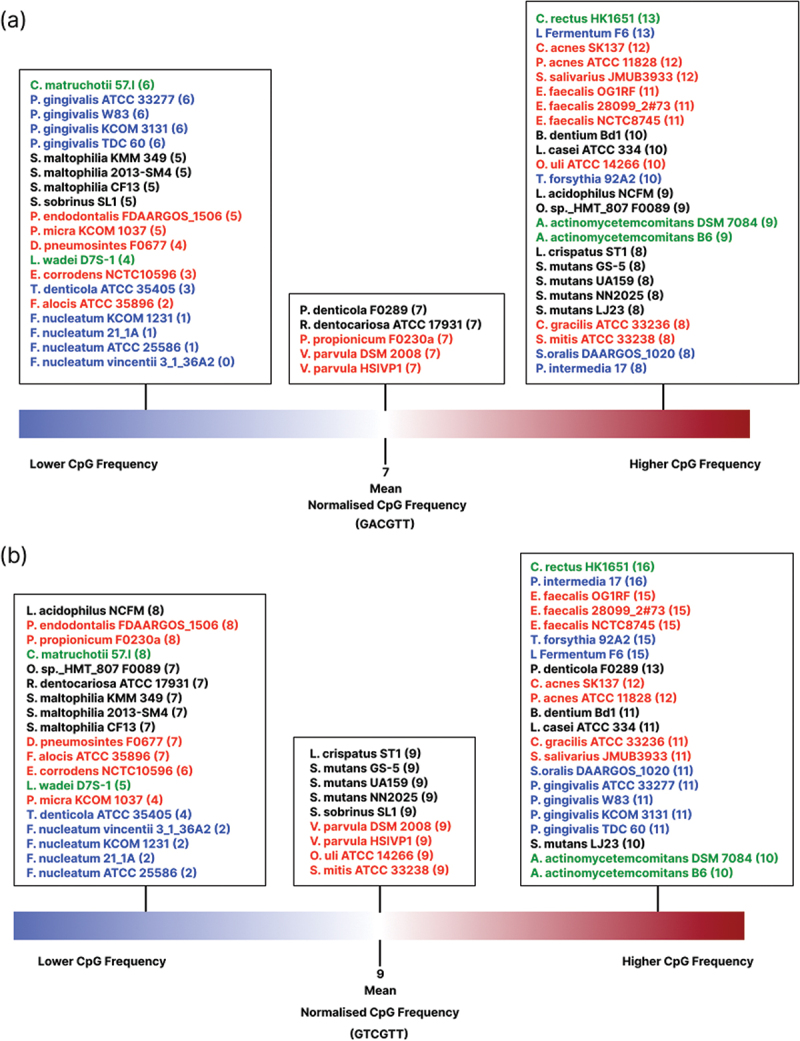
Following normalisation, the frequencies of MRIM (GACGTT) and HIM (GTCGTT), were illustrated. (a) The mean frequency of MRIM (GACGTT) was 7, with 25 bacteria exceeding this mean, 5 had the mean frequency, and 20 bacteria were below the mean. (b) HIM (GTCGTT) mean frequency was found to be 9, with 22 bacteria exhibiting frequency above the mean, 9 possessing the mean frequency, and 19 falling below. Black represents cariogenic bacteria, red represents endodontic bacteria, green represents periodontal bacteria, and blue represents bacteria related to multiple oral diseases.

To account for potential bias influenced by genome size and GC% content, the CpG motif frequencies were normalised, enabling an equitable comparison of the optimum immunostimulatory sequences between bacteria (Figure 3). The normalised mean frequency of the MRIM (GACGTT) was 7, with *Campylobacter rectus* HK1651 and *Limosilactobacillus fermentum* F6 showing the highest frequency for 13 (Figure 3a). Similarly, the mean frequency of the HIM (GTCGTT) was 9, with *C. rectus* HK1651 and *P. intermedia* 17 observed at the highest frequency of 16 (Figure 3b). Nonetheless, *F. nucleatum* strains exhibited the lowest frequency for immunostimulatory sequences.

Our analysis demonstrates that 25 bacteria exhibited a higher than mean frequency of MRIM (GACGTT) (Figure 3a), the majority being bacteria associated with endodontic diseases (e.g. *Enterococcus faecalis* and *Propionibacterium acnes*) and dental caries (e.g. *S. mutans* and *Lacticaseibacillus casei*), each accounting for 9 bacteria. A higher frequency of HIM (GTCGTT) was observed in 22 bacteria (Figure 3b), with 9 bacteria causing multiple oral diseases (e.g. *P. intermedia* and *P. gingivalis*) and 7 causing endodontic diseases (e.g. *E. faecalis* and *P. acnes)*. There were two periodontal bacterial species displayed high frequencies of both MRIM (GACGTT) and HIM (GTCGTT) (*C. rectus* and *Aggregatibacter actinomycetemcomitans)*.

## Discussion

Endodontic infections are characterised by inflammatory reactions involving the cascades of immune response against pathogens, which can result in tissue destruction such as bone loss [29]. Bacteria can modulate the host immune system, activating innate immune cells like neutrophils and macrophages. These activated immune cells produce various pro-inflammatory cytokines, such as IL-1, IL-8, TNF-α, and interferon (IFN)-γ, which then stimulate matrix metalloproteinase (MMPs) and osteoclastic activity, damaging connective tissue and bone [29]. The key mechanism by which bacteria trigger immune response is through various TLR signalling pathways, including TLR-9, which is expressed in multiple types of immune cells, fibroblasts, and both osteoblasts and osteoclasts [30]. Bacterial CpG motifs are recognised as potent immunostimulants targeting the TLR-9 signalling pathway [15]. The immunostimulatory sequences correlate with the GC% content [19]. However, bacterial genomes display variations in GC% content, which is influenced by different environmental factors. For instance, bacteria living in stable environments, such as symbiotic bacteria, tend to lose unnecessary genes and require less energy for survival, resulting in a smaller genome size and a lower GC% content [21].

Our analysis demonstrates that most oral bacteria associated with endodontic diseases, periodontal diseases, and dental caries exhibited medium GC% content (Figure 1a). This finding emphasises the potential role of oral bacterial CpG motifs in triggering host immune response, as GC% content has been shown to have a positive association with the frequency of immunostimulatory sequences (Figure 2). In this study, 60% of oral bacteria associated with endodontic diseases, periodontal diseases, dental caries, and multiple oral diseases exhibited medium GC% content (*Mdn* = 44). There was no significant difference in GC% content across bacterial groups (*p* = 0.66, Figure 1b). These results suggest that although oral bacteria niches show variation in different diseases, the core members contributing to oral diseases present similar GC% content in their genomes. This similarity might be due to the overlapping profiles between endodontic and periodontal bacterial communities [31]. Moreover, endodontic infection shares a similar location and environment with dental caries, particularly deep carious lesions, both of which are characterised by oxygen-limited conditions [32]. Therefore, these might explain the similarity of GC% content of oral bacteria between groups.

Our analysis indicates that the frequency of CpG motifs increases with higher GC% content and larger genome size (Figure 2). This observation aligns with the expectation that bacteria harbouring larger genomes and higher GC% content naturally possess a higher occurrence of CpG motifs. Therefore, further analysis was performed to normalise the frequency count for equitable comparison among bacteria. Following normalisation, our analysis reveals that the majority of bacteria related to endodontic diseases (9/14) and dental caries (9/10) possessed higher-than-mean MRIM (GACGTT) frequency. Moreover, endodontic bacteria also displayed a high frequency of HIM (GTCGTT) (7/14). These results suggest that core bacteria implicated in endodontic diseases may elicit intense immune responses during infection, potentially contributing to the acute pain seen in pulpitis associated with increased pressure. Notably, key periodontal pathogens like *A. actinomycetemcomitans* and *C. rectus*, were also observed to have a high frequency of immunostimulatory CpG motifs. To our knowledge, there is no strong evidence suggesting the bacteria species with low frequency of CpG motifs in their genome can avoid immune stimulation for evading the host immune responses.

Our study found those bacteria related to primary endodontic infections, such as *C. gracilis* and *P. acnes*, exhibited an above mean frequency of MRIM (GACGTT) and HIM (GTCGTT). *C. gracilis*, a Gram-negative anaerobe, has been reported to be associated with primary endodontic infections [33]. While the pathophysiological mechanism involved in endodontic diseases is complex, a high frequency of CpG motifs might be a virulence factor triggering inflammation. *P. acnes* is a Gram-positive anaerobe that colonises the skin and oral cavity as part of the normal microbiota [34]. One study reported *P. acnes* as the most prevalent species identified in primary endodontic infections [35]. A high-frequency count of immunostimulatory sequences might explain its pathogenicity to activate TLR-9 signalling, leading to IFN-γ release [36] and potentially contributing to pulpitis and apical periodontitis.

*E. faecalis*, a Gram-positive anaerobe, has been significantly associated with persistent endodontic infections [37]. Despite *E. faecalis* infection often being asymptomatic, it was regarded as a significant cause of endodontic treatment failure [38]. The aggregation substance of *E. faecalis* can induce the release of TNF-α and IFN-γ, leading to bone resorption. Furthermore, *E. faecalis* is known for its antimicrobial resistance and ability to evade the host immune system. In our study, all three strains of *E. faecalis* exhibited a high occurrence of MRIM (GACGTT, 11) and HIM (GTCGTT, 15), suggesting a potential role for CpG motifs in its pathogenicity.

*C. rectus*, a Gram-negative anaerobe, is established as the dominant species implicated in periodontitis [39]. It has been demonstrated that *C. rectus* is a potent immunostimulatory bacterium, capable of increasing mRNA levels of IL-6, IL-8, and TNF-ɑ, which are pro-inflammatory cytokines involved in the pathogenesis of periodontitis [40,41]. Our results indicate that *C. rectus* showed the highest frequency count for MRIM (GACGTT, 13) and HIM (GTCGT, 16). This observation might explain the strong pathogenicity of *C. rectus* in triggering the host immune response. Similarly, *A. actinomycetemcomitans*, a dominant Gram-negative anaerobe highly associated with rapidly progressing periodontitis [42], also exhibited a high frequency of both MRIM (GACGTT, 10) and HIM (GTCGTT, 10). Such grades of periodontitis are characterised by an intense host immune response against bacterial infection, yielding rapid tissue destruction and bone loss [43]. *A. actinomycetemcomitans* possesses several virulence factors that activate the immune system, such as TLR-4 signalling induced by LPS and increased production of IL-1β and IL-18 in monocytes by leukotoxin [44]. The observed high frequency of immunostimulatory CpG motifs in *A. actinomycetemcomitans* could be a further potent virulence factor to activate the host immunity.

Black-pigmented Gram-negative anaerobes, including *P. intermedia and P. gingivalis*, are recognised to be involved in the pathogenesis of endodontic and periodontal diseases. Our study found that these bacteria possessed high counts of CpG motifs, with *P. intermedia* exhibiting the highest frequency of HIM (GTCGTT, 16) and a high frequency of MRIM (GACGTT, 8). Similarly, all four strains of *P. gingivalis* in our study showed a high frequency of HIM (GTCGTT, 11), but a lower frequency of MRIM (GACGTT, 6). These bacteria are frequently isolated from periapical and periodontal abscesses [45]. *P. intermedia* and *P. gingivalis* are well-established pathogens known to enhance the production of IL-1β, IL-8, TNF-α, and MMPs production, ultimately leading to tissue breakdown and bone resorption [46,47]. Additionally, *T. forsythia*, a Gram-negative anaerobe, showed a high frequency of MRIM (GACGTT, 10) and HIM (GTCGTT, 15). The prevalence of *T. forsythia* increased in subgingival plaque of periodontitis patients compared to healthy controls [48]. One study suggested a link between *T. forsythia* and pain and swelling in endodontic diseases [49]. *T. forsythia* can invade and degrade host tissue through protease activity, contributing to endodontic diseases and periodontal diseases [50]. Interestingly, one study also demonstrated that bacterial DNA from *P. gingivalis* and *T. forsythia* can enhance the production of pro-inflammatory cytokines *via* TLR-9 signalling pathway in human monocytic cells, suggesting a potential role of bacterial CpG motifs in host immune modulation [51]. Conversely, *F. nucleatum*, a Gram-negative anaerobe, demonstrated the lowest GC% content and the least frequent occurrence of CpG motifs in our study. However, *F. nucleatum* is a dominant pathogen in endodontic diseases and periodontal diseases [52]. While CpG can activate the TLR-9 signalling pathway, the *F. nucleatum* cell wall can stimulate the immune response by upregulating pro-inflammatory cytokines *via* NF-κB pathway activation [53]. Moreover, *F. nucleatum* can induce an immune response *via* TLR-4, further promoting inflammation [52]. These findings highlight the complexity of immune response pathways contributing to the inflammatory process in endodontic and periodontal diseases.

*L*. *fermentum*, a Gram-positive anaerobe frequently isolated from carious lesions [54], displayed the highest frequency of MRIM (GACGTT, 13) and showed a high frequency of HIM (GTCGTT, 15). While *L. fermentum and* other Lactobacilli are dominant in carious lesions [55], they require co-colonisation with pathogens like *S. mutans* to cause caries [56]. Our study also reveals that *S. mutans* and *L. casei*, key pathogens in cariogenesis, demonstrated a high frequency of MRIM (GACGTT) and HIM (GTCGTT). Although dental caries is primarily a demineralisation disease, the inflammatory response in dental pulp remains pivotal in disease progression. Bacteria can stimulate the production of pro-inflammatory cytokines (e.g. IL-6 and IL-8), leading to the infiltration of immune cells (e.g. dendritic cells and macrophages) within the pulp tissue [57]. The ongoing inflammatory process can ultimately result in pulpitis. Therefore, our results suggest a potential role for cariogenic bacterial CpG motifs in triggering the immune response in dental pulp during caries progression. This mechanism might involve the diffusion of CpG through dentinal tubules, subsequently activating host immune cells within the dental pulp.

This present study emphasises the importance of CpG motifs within the genomes of oral bacteria as a potential virulence factor contributing to endodontic diseases, periodontal diseases, or dental caries. The route of bacterial CpG immune activation is through TLR-9 recognition in various cell types. Limitations in this study were the selection of bacterial strains, which was based on the previous literature and the availability of relevant databases, as most studies reported bacterial prevalence at a broader taxonomic level. However, key pathogenic strains were selected to the best extent possible. Moreover, we propose further investigation into the *in vitro* and *in vivo* experiments of oral bacterial CpG motifs interactions in biological models to characterise the effects of CpG frequency based on these findings. A comprehensive insight of these interactions is essential to elucidate the precise effect by which bacterial CpG motifs contribute to oral disease progression, thereby facilitating the invention of novel therapeutic targets.

## Conclusion

Bacterial CpG motifs are recognised as potent immunostimulants that activate the immune response through the TLR-9 signalling pathway, consequently promoting inflammation. In our study, most of oral bacteria associated with endodontic diseases, periodontal diseases, and dental caries exhibit medium GC% content. The GC% is known to correlate with the occurrence of immunostimulatory sequences capable of triggering immune responses. These characteristics underscore the influence of these bacteria in the inflammatory process of oral diseases. Therefore, bacterial CpG motifs might play a role in oral disease progression, represent how differences in bacterial flora can affect disease risk and represent a new line of investigation to combat inflammation in odontogenic diseases.

## Supplementary Material

Supplementary_CpG.xlsx

## Data Availability

The data underlying this article are available in the article and in its online supplementary material. The genome data are available in the GenBank Nucleotide Database at [www.ncbi.nlm.nih.gov] and can be accessed with accession numbers.
